# Comparison of Chlorophyll and Bacteriochlorophyll Ultrafast Transient Absorption Spectra and Kinetics

**DOI:** 10.3390/molecules31060939

**Published:** 2026-03-11

**Authors:** Arjun Krishnamoorthi, Negar Karpourazar, Keyvan Khosh Abady, Peter M. Rentzepis

**Affiliations:** Department of Electrical and Computer Engineering, Texas A&M University, College Station, TX 77843, USA

**Keywords:** chlorophylls, bacteriochlorophylls, photosynthesis, transient absorption, ultrafast spectroscopy

## Abstract

Oxygenic and anoxygenic photosynthesis are initiated through the absorption of light by chlorophyll and bacteriochlorophyll photosynthetic pigments, respectively, which function as light-harvesting (antenna) and redox pigments on the photosynthetic membrane that trap and convert the absorbed optical energy into chemical energy. While several studies have characterized the ultrafast spectra, kinetics, and structures of the light-harvesting and reaction center complexes that contain the photosynthetic pigments, a detailed understanding of how the ultrafast excited-state dynamics vary across different photosynthetic pigments is lacking. Such information is critical in understanding the molecular mechanisms of both artificial and natural photosynthetic systems. In this study, we conducted ultrafast time-resolved absorption spectroscopy on chlorophyll and bacteriochlorophyll photosynthetic pigments at room temperature to directly compare the spectra and kinetics of their transient, excited electronic states formed following photon absorption. The recorded ultrafast spectral and kinetic data, spanning the femtosecond to sub-microsecond timescales, show interesting similarities and differences between these two distinct types of photosynthetic pigments. These experimental results help clarify the relationship between photosynthetic pigment structure and the resultant ultrafast processes in the oxygenic and anoxygenic photosynthetic reaction mechanisms.

## 1. Introduction

Chlorophylls and bacteriochlorophylls are the two primary types of photosynthetic pigments that enable the light reactions of oxygenic and anoxygenic photosynthesis, respectively [1,2,3]. While chlorophylls are commonly found in oxygen-producing species such as plants, algae, and cyanobacteria, bacteriochlorophylls are instead vital components of phototrophic, colored bacteria such as *Rhodobacter sphaeroides* and *Blastochloris viridis* that can grow anaerobically [2]. The general mechanism of photosynthesis utilizes chlorophylls or bacteriochlorophylls as both light-harvesting (antenna) pigments that capture the incident optical energy and redox pigments that facilitate a series of sequential electron transfers for converting the absorbed optical energy into chemical energy [3,4,5,6]. In conjunction with accessory pigments such as carotenoids [7], chlorophylls and bacteriochlorophylls function as antenna pigments in peripheral light-harvesting complexes (LHCs) [3] that absorb, harvest, and rapidly transfer the absorbed optical energy to the core reaction center (RC) pigment-protein complex, where light-induced charge separation across the photosynthetic membrane begins. The concomitant generation of a proton gradient and membrane potential [1,2,4,5] drives the synthesis of ATP, which fuels the subsequent dark reactions that produce photosynthetic reaction products. While the precise structure and mechanism of a photosynthetic system may vary considerably among different organisms [3], the general features of a light-harvesting complex and reaction center, which both contain photosynthetic pigments like chlorophylls and bacteriochlorophylls, are common. In fact, it has been shown that RC complexes found in photosystems I and II of oxygenic photosynthetic systems are structurally similar to those found in photosynthetic anaerobic bacteria, with both types of photosynthetic systems having remarkably high quantum efficiencies that are nearly unity [5].

Over the past several decades, substantial research has been directed toward characterizing the ultrafast processes in photosynthesis by means of steady-state and time-resolved optical spectroscopy. Initial steady-state studies [8,9] of the photosynthetic pigments revealed the characteristic broad, diffuse short-wavelength and long-wavelength absorption bands located near the blue and red spectral regions, respectively, that are common to all chlorophyll and bacteriochlorophyll photosynthetic pigments [2]. Subsequently, the development of flash and laser photolysis [10,11] enabled studies of the chlorophyll and bacteriochlorophyll long-lived first excited electronic triplet energy states and corresponding excited triplet-state absorption spectra [4,12,13,14]. The advent of mode-locked lasers and pump–probe spectroscopic techniques [15,16] was further instrumental in resolving ultrafast molecular processes in photosynthesis. Picosecond optical pulses were used to excite chlorophyll [17,18,19] and determine the ultrafast transient absorption spectra and lifetime of its first excited electronic singlet energy state, which is rapidly occupied and subsequently decays to either the ground singlet state, through both internal conversion and fluorescence emission, or lower-lying excited triplet state by means of intersystem crossing. Several studies [19,20,21,22,23] have also explored the solvent dependences of the singlet and triplet excited-state spectral and kinetic properties. In addition to these studies on isolated photosynthetic pigments, ultrafast time-resolved spectroscopic studies of bacterial RC complexes [5,24,25,26,27,28,29,30] revealed the key molecular components that are involved in the light reactions of bacterial photosynthesis, supporting a mechanism whereby a bacteriochlorophyll dimer, following the occupation of its first excited singlet state through either photon absorption or energy transfer, transfers an electron via an accessory bacteriochlorophyll monomer to a bacteriopheophytin. The excited bacteriochlorophyll dimer thus serves as the primary electron donor, while the bacteriopheophytin is the primary electron acceptor, that initiates a series of sequential electron transfers necessary for creating a membrane potential [5]. Femtosecond transient absorption spectroscopy has revealed that this primary event in bacterial photosynthesis, namely the photo-oxidation of a bacteriochlorophyll dimer and concomitant photo-reduction of bacteriopheophytin, occurs within a few picoseconds [28], while the subsequent electron transfer from bacteriopheophytin to the secondary electron acceptor, a quinone, occurs in hundreds of picoseconds [26]. The conformational dynamics of this initial event in photosynthesis, which facilitate the formation and stabilization of a charge-separated state, have been studied in microcrystals of a bacterial RC with atomic spatial resolution through time-resolved femtosecond crystallography using an X-ray free-electron laser [31]. Ultrafast time-resolved spectroscopic measurements [32,33,34,35,36,37,38] have also been performed on RC complexes comprising photosystems I and II, and the data suggest that an analogous electron transfer mechanism occurs in oxygenic photosynthetic systems with a chlorophyll species functioning as the primary electron donor [3,6,39,40].

There has been long-standing, fundamental interest in the characterization of the spectral properties of photosynthetic pigments, namely the relationship between pigment structural features and the corresponding vibronic transitions. Such information is paramount for understanding, designing, and modifying both natural and artificial photosynthetic systems. Structurally, all photosynthetic pigments are metalloporphyrin compounds that exhibit characteristic singlet-singlet (S_0_→S_n_) electronic absorption bands in the ~350–450 nm (Soret or B bands) and ~550–800 nm (Q bands) spectral regions that are described by the “four-orbital” model for the two highest occupied molecular orbitals (HOMOs) and two lowest unoccupied molecular orbitals (LUMOs) [2,41]. Differences in the degree of conjugation in the pigment macrocycle, along with varying terminal substituents, result in three general types of photosynthetic pigments: porphyrin, chlorin, and bacteriochlorin [2,42,43]. Chlorophyll *a* (Chl *a*) is the primary chlorin-type pigment used for oxygenic photosynthesis, whereas bacteriochlorophyll *a* (BChl *a*) is the major bacteriochlorin-type pigment found in anoxygenic photosynthetic bacteria [2,6]. Substantial work has been conducted in recording and comparing the ground-state singlet and excited-state triplet spectral and kinetic properties [23,42,44,45], along with the fluorescence and phosphorescence spectra and lifetimes [20,43,46], of various types of natural and chemically modified photosynthetic pigments. The singlet-triplet energy gaps, in addition to an energy gap law for describing the triplet-state lifetime and energy, have been extensively characterized for both chlorophylls and bacteriochlorophylls [42,43]. These studies have demonstrated that a rather strong correlation exists between the photosynthetic pigment type and the corresponding excited-state properties. Substitutions in the central metal atom, which is usually Mg, bound to the pigment macrocycle have also been shown to induce both ground-state and excited-state spectral and kinetic shifts [23,47]. Detailed knowledge of the pigment triplet electronic structure is particularly important for understanding and engineering the function of carotenoids, which play key roles in photosynthesis as both accessory pigments and triplet-state quenchers [7,48]. While many comparative studies on photosynthetic pigments have primarily addressed excited triplet-state properties, and thus probe on timescales of nanoseconds or longer following optical excitation, relatively few studies [47,49,50,51,52] have investigated spectral and kinetic differences on timescales prior to excited triplet-state occupation. Consequently, substantially less is known about how the ultrafast, femtosecond to picosecond, time-resolved spectra and kinetics differ between the various photosynthetic pigments. Such ultrafast timescales are crucial for studying the pigment excited singlet states involved in the initial steps of photosynthesis. In addition to extending our understanding about differences in the oxygenic and anoxygenic photosynthetic reaction mechanisms, this information would be valuable for the creation of artificial photosynthetic systems [53,54,55].

In this study, we conducted ultrafast time-resolved absorption spectroscopic experiments on the photosynthetic pigments Chl *a* and BChl *a* on the femtosecond to sub-microsecond timescales at room temperature to compare directly the transient spectra and lifetimes of their excited states. Such an extensive probe temporal range allowed us to observe the optical pump-induced ultrafast dynamics of excited singlet-state occupation, internal conversion, intersystem crossing to the lower-lying excited triplet state, and ultimately relaxation back to the singlet ground state. This is in marked contrast to previous comparative studies [42,43] that have primarily examined triplet-state properties by pumping and probing on significantly longer timescales. Thus, the primary novelty of our study is the direct comparison of the primary photophysics of the Chl *a* and BChl *a* photosynthetic pigments on the ultrafast timescale, which has been relatively unexplored in the literature. Our experimental results provide a direct comparison of the ultrafast responses of chlorin-type (Chl *a*) and bacteriochlorin-type (BChl *a*) photosynthetic pigments due to optical excitation in the B and Q steady-state absorption bands, and we have observed several interesting spectral and kinetic differences between the two pigment types. This spectroscopic and kinetic comparison of distinct photosynthetic pigments over a broad range of timescales, encompassing the transient spectra and kinetics of both their excited singlet and triplet electronic states, is the major contribution of this paper. These findings extend our knowledge about the relationship between the photosynthetic pigment type and corresponding ultrafast optical properties. The significance of this study is an improved understanding of the basic ultrafast photophysics of the two major biological pigments involved in oxygenic and anoxygenic photosynthesis.

## 2. Materials and Methods

### 2.1. Photosynthetic Pigments and Steady-State Spectra

The photosynthetic pigments Chl *a* (Product Number: 96145) and BChl *a* (Product Number: B5906) were purchased from Sigma-Aldrich (MilliporeSigma, St. Louis, MO, USA) and used without further purification. Each pigment was solubilized by gently stirring the pigment in spectroscopic-grade methanol at ~4 °C in the dark overnight. This resulted in stock solutions of Chl *a* and BChl *a* in methanol with estimated concentrations of ~1 mg/mL each. For the steady-state and time-resolved spectroscopic measurements, dilute solutions were prepared from these stock solutions to have a typical steady-state optical density (OD) of ~1–1.50 (for a 2 mm or 10 mm path length, depending on the experiment) at the corresponding pump wavelength employed in the experiment. All photosynthetic pigment solutions were kept at ~−20 °C in the dark until use.

**Figure 1:**
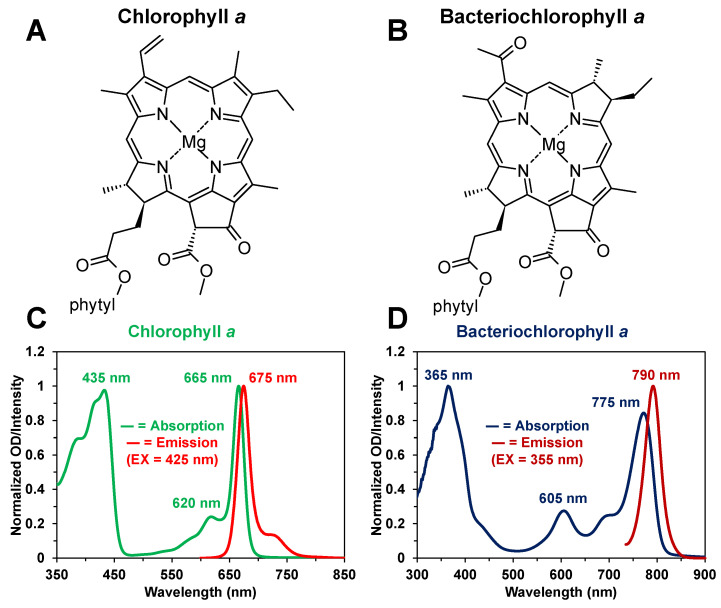
Chemical structures of the photosynthetic pigments (**A**) Chl *a* and (**B**) BChl *a* compared in this study. The chemical structures were downloaded from PhotochemCAD [56,57,58] and modified for presentation in ChemSketch Freeware software (ChemSketch Freeware, version 2025.2.0, Advanced Chemistry Development, Inc. (ACD/Labs), Toronto, ON, Canada, [59]). The corresponding normalized steady-state absorption and fluorescence spectra of (**C**) Chl *a* and (**D**) BChl *a* in methanol are also provided with the approximate band maxima noted.

The steady-state absorption and fluorescence spectra of the photosynthetic pigments were recorded using a Shimadzu 1601 UV-Vis spectrophotometer and Shimadzu RF-6000 fluorophotometer (Tokyo, Japan), respectively. The fluorescence spectra were recorded with an emission bandwidth (resolution) of ~3 nm. Quartz cuvettes with either a 2 mm or 10 mm path length, depending on the experiment, were employed for the steady-state spectral measurements. The recorded steady-state absorption spectra of Chl *a* and BChl *a* displayed good consistency in both band maxima and shape across the range of concentrations used for the femtosecond, picosecond, and nanosecond transient absorption experiments. The chemical structures of Chl *a* and BChl *a*, in addition to their corresponding steady-state absorption and fluorescence spectra in methanol, are shown in Figure 1.

### 2.2. Femtosecond Transient Absorption System and Experiments

**Figure 2:**
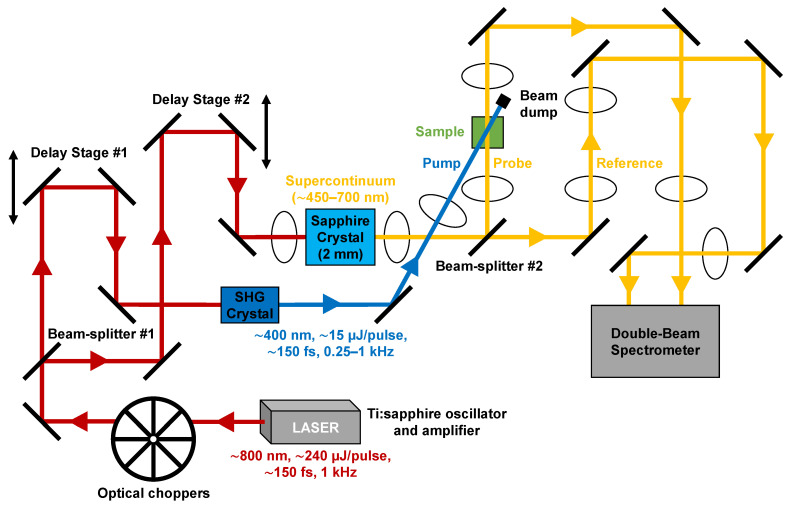
Schematic diagram of the femtosecond transient absorption experimental system.

Our femtosecond transient absorption experimental system, as shown in Figure 2, consists of a regeneratively mode-locked Ti:sapphire oscillator (Spectra-Physics, Milpitas, CA, USA, Model: Tsunami) and chirped pulse amplifier (Spectra-Physics, Milpitas, CA, USA, Model: Spitfire) that produce ~800 nm, ~150 fs fundamental pulses with a pulse energy of ~240 µJ at a repetition rate of 1 kHz. A series of optical choppers in the fundamental beam path allows the repetition rate to be reduced to as low as 250 Hz. Most of the fundamental output is passed through a nonlinear SHG crystal to generate the corresponding second harmonic at ~400 nm with a maximum SHG pulse energy of ~15 µJ. This SHG output is subsequently attenuated and used for optically pumping the photosynthetic pigments in their respective Soret absorption bands. The remaining portion of the ~800 nm fundamental beam is focused into a thin, ~2 mm, sapphire crystal plate to generate a broad supercontinuum (~450–700 nm with filters) that is subsequently divided into probe and reference beam paths for interrogating the sample at a given pump–probe delay time and monitoring variations in the supercontinuum, respectively. The precise pump–probe delay time is determined by means of linear translation delay stages that temporally synchronize the pump and probe pulses, which are spatially overlapped in the sample cell, with delay times up to a few hundred picoseconds. The probe and reference beam spectra, with and without the pump pulse, are detected and recorded by means of a double-beam spectrograph (Acton Research Corporation, Acton, MA, USA, Model: SpectraPro-150) attached to a water-cooled CCD camera (Princeton Instruments, Trenton, NJ, USA, Model: DPDA-1024), which facilitates the computation of ∆OD spectra as a function of pump–probe delay time. This ultrafast pump–probe experimental system has sufficient sensitivity to detect transient absorption signals of a few mOD in magnitude with a temporal resolution of ~350 fs based on the FWHM of the corresponding pump–probe cross-correlation function. The pump–probe cross-correlation function was estimated based on the stimulated Stokes Raman scattering signal from distilled water recorded at ~460 nm [60,61].

For the femtosecond transient absorption experiments, samples of Chl *a* and BChl *a* in methanol were concentrated to have a steady-state OD of ~1 (2 mm path length) at the ~400 nm pump wavelength, and ~2 mL of the sample was placed inside a 2 mm quartz flow cell. The femtosecond laser system was operated in pulsed mode at a repetition rate of 500 Hz with an ~400 nm pump pulse energy of ~750 nJ and pump beam diameter of ~500 µm at the sample. To avoid photobleaching effects, the sample solution was continuously circulated by means of a closed-loop flow system that employs a small peristaltic pump (Kamoer, Shanghai, China, Model: KCP PRO2). All experiments were conducted at room temperature under aerobic conditions and in the dark with dim green background light. Steady-state absorption spectra of the samples were recorded before and after each experiment to confirm no significant photodegradation occurred.

### 2.3. Picosecond Transient Absorption System and Experiments

**Figure 3:**
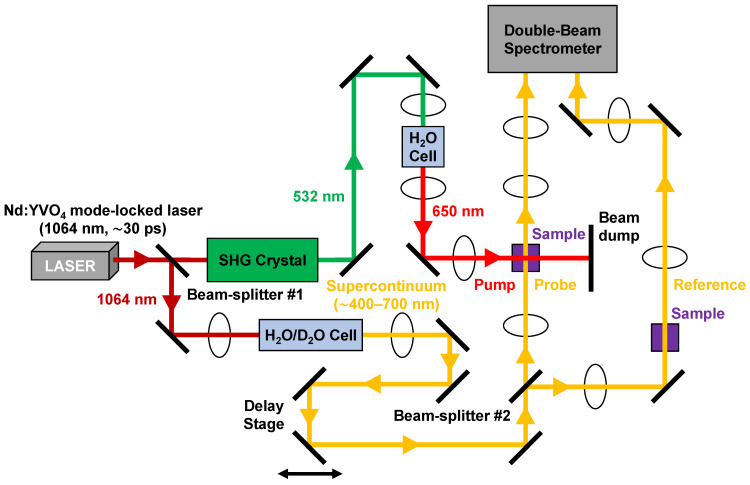
Schematic diagram of the picosecond transient absorption experimental system.

Our picosecond transient absorption experimental system, as shown in Figure 3, has been described previously [62,63,64]. Briefly, part of the ~1064 nm fundamental output of a passively mode-locked Nd:YVO_4_ picosecond oscillator (Ekspla, Vilnius, Lithuania, Model: PL2230) propagates through a nonlinear SHG crystal for the generation of the corresponding second harmonic output at ~532 nm. Subsequently, this picosecond harmonic pulse is focused into a 10 cm H_2_O cell to produce stimulated Stokes Raman output at ~650 nm for optically pumping the photosynthetic pigments in their respective Q absorption bands. The ~650 nm pump pulse width is ~30 ps with a pulse energy of ~1 mJ and pump beam diameter of ~1.5 mm at the sample. The residual portion of the ~1064 nm fundamental output is focused into a 20 cm H_2_O/D_2_O cell to generate a broadband, visible region supercontinuum (~400–700 nm with filters) that is subsequently divided into probe and reference beams. The probe beam is focused and spatially overlapped with the stimulated Stokes Raman pump pulse at the sample, while the reference beam is used to correct for fluctuations in the supercontinuum. Both the probe and reference beams are collected and focused into a spectrograph (Acton Research Corporation, Model: SpectraPro-150) that is coupled with a TE-cooled 2-D CCD camera (Princeton Instruments, Model: PIXIS:400) maintained at −75 °C. The probe and reference spectra are recorded with and without the pump pulse, which enables measurement of the ∆OD spectrum at a specific pump–probe delay time. The pump and probe pulses are temporally synchronized by means of a linear translation delay stage, which allows the probe pulse to be delayed with respect to the pump pulse for delay times up to a few nanoseconds.

For the picosecond transient absorption experiments, samples of Chl *a* and BChl *a* in methanol were concentrated to have a steady-state OD of ~1.25–1.5 (10 mm path length) at the ~650 nm pump wavelength. A portion of the sample was placed in a 10 µL microchannel quartz cuvette with a 10 mm probe path length, 1 mm pump path length, and cuvette cap. The picosecond pump laser was operated in single-shot mode. At each pump–probe delay step, up to 8 pump–probe shots were performed on a given sample aliquot. The sample was replaced with a fresh sample aliquot at each pump–probe delay step to avoid sample evaporation and/or photobleaching effects. All experiments were conducted at room temperature under aerobic conditions and in the dark with dim green background light. Under these conditions, the recorded ∆OD spectra for a given sample aliquot were highly consistent, in both peak amplitude and shape, across multiple pump laser shots.

### 2.4. Nanosecond Transient Absorption System and Experiments

**Figure 4:**
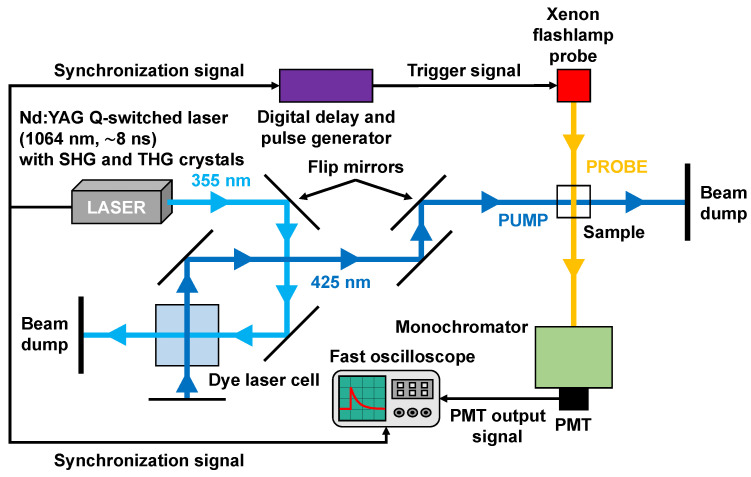
Schematic diagram of the nanosecond transient absorption experimental system. Note that either the ~355 nm THG or ~425 nm dye laser output is used as the pump pulse, depending on the sample.

Our nanosecond transient absorption experimental system, as shown in Figure 4, has been described previously [62,63,64,65]. Briefly, the third harmonic, ~355 nm, output of a Q-switched Nd:YAG nanosecond laser system (New Wave Research, Fremont, CA, USA, Model: Tempest-20) is used, along with a dye laser cell, for optically pumping the photosynthetic pigments in their respective Soret absorption bands. For the Chl *a* time-resolved experiments, a homebuilt dye laser system composed of the laser dye stilbene 420 (Exciton, Lockbourne, OH, USA) in methanol (~0.3 mg/mL in a 1 cm quartz cuvette) is used to generate ~8 ns (FWHM) pump pulses centered at ~425 nm with a typical pulse energy of ~2 mJ. The ~425 nm dye laser emission was focused with a cylindrical lens to an ~5 mm length and ~1 mm width at the sample. For the BChl *a* time-resolved experiments, the ~8 ns (FWHM) THG pulses at ~355 nm are directly used as pump pulses with a typical pulse energy of ~8 mJ and pump beam diameter of ~5 mm at the sample. The optical probe is a Xenon flashlamp module whose output emission, ~100 µs (FWHM) in pulse duration, is focused and spatially overlapped with the pump pulse in the sample cell. Temporally, the practically constant region, ~4 µs in duration, of peak flash intensity is synchronized with the pump laser pulse impinging on the sample. Transient variations in the Xenon flashlamp probe intensity are recorded in time by means of a photomultiplier tube (Hamamatsu, Bridgewater, NJ, USA, Model: R928) coupled to the output of a monochromator (Jarrell Ash, Boston, MA, USA, Model: 82-410), which is positioned after the sample cell, that can be manually scanned across different probe wavelengths. A fast oscilloscope (Tektronix, Beaverton, OR, USA, Model: TDS3052B), with an input impedance of 50 Ω and corresponding PMT-oscilloscope rise time of a few nanoseconds, is used to measure the photomultiplier voltage as a function of time. Recording the photomultiplier voltage before and after the “time zero” delay step, when the pump pulse is temporally coincident with the probe pulse at the sample, facilitates the measurement of ∆OD as a function of the pump–probe delay time. This provides the transient ∆OD kinetics at a specific probe wavelength, and scanning the monochromator thus enables computation of the transient ∆OD spectra for pump–probe delays in the range of a few nanoseconds up to a few microseconds.

For the nanosecond transient absorption experiments, samples of Chl *a* and BChl *a* in methanol were concentrated to have a steady-state OD of ~1 (10 mm path length) at the ~425 nm and ~355 nm pump wavelengths, respectively. A portion of the sample was placed in a 10 µL microchannel quartz cuvette with a 10 mm probe path length, 1 mm pump path length, and cuvette cap. The nanosecond pump laser was operated in single-shot mode. The sample was replaced with a fresh sample aliquot after every 3 to 5 pump laser shots, depending on the experiment, and at every new probe wavelength to avoid sample evaporation and/or photobleaching effects. All experiments were conducted at room temperature under aerobic conditions and in the dark with dim green background light. Under these conditions, the recorded ∆OD kinetic traces for a given sample aliquot were highly consistent, in both peak amplitude and shape, across multiple pump laser shots.

### 2.5. Data Analysis

For the femtosecond transient absorption experiments, the recorded sets of probe and reference spectra (with and without the pump pulse) were used to compute the corresponding set of ΔOD spectra at the various pump–probe delay times. These ΔOD spectra calculations were performed directly in the corresponding WinSpec software (Princeton Instruments, Version: WinSpec/32). The time zero delay step, when the pump and probe pulses are temporally coincident at the sample, was estimated based on the rise in the ΔOD signal at probe wavelengths of ~465 nm for Chl *a* and ~525 nm for BChl *a*, which correspond to their respective ΔOD band maxima. Background subtraction was performed using the ΔOD spectra recorded well before time zero. We note that the femtosecond transient absorption data in this study were not corrected for dispersion of the probe pulse, which is a well-documented phenomenon [61,66] in ultrafast experiments that results in the apparent bathochromic shift of the ∆OD band maximum within the first picosecond or so following excitation. This phenomenon, however, does not impact the sample dynamics at the detected probe wavelengths, which is the primary interest in this study. Linear interpolation of the recorded ΔOD datasets was performed in MATLAB (Version: R2024b) to produce corresponding 2D contour plots.

For the picosecond transient absorption experiments, the ΔOD spectra were computed and processed using similar methods as for the femtosecond datasets. For the nanosecond transient absorption experiments, the ΔOD kinetic trace at each probe wavelength was computed based on the recorded probe intensity before and after the arrival of the pump pulse at time zero. The determination of time zero, along with the single-term exponential fittings (with or without a constant offset, depending on the experiment) of the ∆OD kinetics at all the scanned probe wavelengths, was performed through a custom-written MATLAB program. To minimize the effect of the PMT instrument response function, the ∆OD kinetic exponential fit at each probe wavelength was computed using only the subset of data points recorded after a fixed time offset relative to time zero. The calculated set of ∆OD kinetic exponential fits, across all probe wavelengths, was then used to derive the corresponding ∆OD spectra at various pump–probe delays.

Single-term exponential fittings of the femtosecond, picosecond, and nanosecond ΔOD kinetics were performed in MATLAB through the Curve Fitting Toolbox. For the femtosecond time-resolved data, the uncertainties in the reported time constants were computed based on the corresponding 90% confidence intervals for the fit parameter. For the picosecond and nanosecond time-resolved data, uncertainties were estimated based on the standard deviation of the time constants computed across multiple pump laser shots.

## 3. Results and Discussion

### 3.1. Femtosecond Transient Absorption Spectra and Kinetics

**Figure 5:**
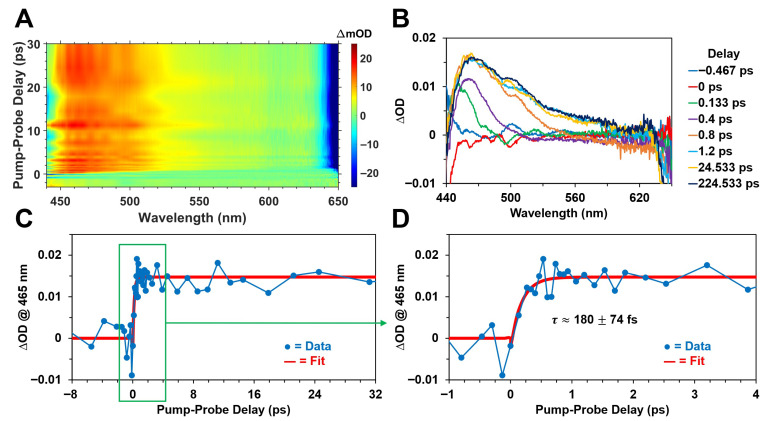
Femtosecond transient absorption spectra and kinetics of Chl *a* in methanol following optical excitation with ~400 nm, ~150 fs pulses at a 500 Hz repetition rate with ~750 nJ/pulse. (**A**) 2D contour plot of the ΔOD signal, displaying the ultrafast formation of a broad, diffuse excited-state absorption band with peak magnitude at ~465 nm, along with a negative band near 650 nm that is attributed to a combination of ground-state bleach and stimulated emission. Note that the ΔOD dataset is not corrected for dispersion of the probe pulse. (**B**) Femtosecond transient absorption spectra at various pump–probe delay times. (**C**) Excited-state formation kinetics monitored at the ~465 nm ∆OD band maximum, illustrating the (**D**) ultrafast formation of the first excited singlet state within hundreds of femtoseconds.

**Figure 6:**
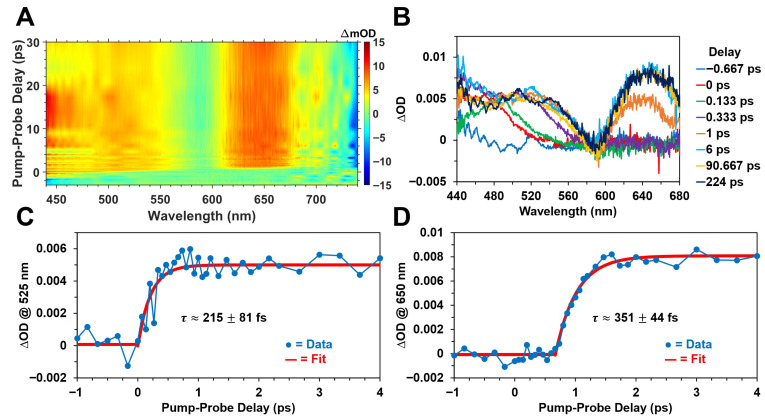
Femtosecond transient absorption spectra and kinetics of BChl *a* in methanol following optical excitation with ~400 nm, ~150 fs pulses at a 500 Hz repetition rate with ~750 nJ/pulse. (**A**) 2D contour plot of the ΔOD signal, displaying the ultrafast formation of broad, diffuse excited-state absorption band maxima with peak magnitudes at ~525 nm and ~650 nm, along with a negative band near 740 nm that is attributed to a combination of ground-state bleach and stimulated emission. Note that the ΔOD dataset is not corrected for dispersion of the probe pulse. (**B**) Femtosecond transient absorption spectra at various pump–probe delay times. Excited-state formation kinetics monitored at the (**C**) ~525 nm and (**D**) ~650 nm ∆OD band maxima, illustrating the ultrafast formation of the first excited singlet state within hundreds of femtoseconds.

Figure 5 and Figure 6 display the femtosecond transient absorption spectra and kinetics of Chl *a* and BChl *a* in methanol, respectively, following optical excitation with ~400 nm, ~150 fs pulses. In agreement with previous ultrafast time-resolved absorption studies [17,18,47,67,68,69,70] on chlorophyll and bacteriochlorophyll pigments, we observed the ultrafast formation of broad, diffuse excited-state absorption bands in the visible spectral region following pulsed excitation for both photosynthetic pigments. We attribute the formation of these absorption bands to the ultrafast occupation of the first (lowest allowed) excited electronic singlet energy state, S_1_, which is the primary excited state that participates in both energy and charge (electron) transfer in photosynthesis [2,3]. The femtosecond transient absorption datasets shown in Figure 5 and Figure 6 are not corrected for dispersion of the probe pulse, which results in the progressive redshift of the ∆OD band maximum within about a picosecond following excitation. Although this results in a wavelength-dependent time zero curvature in the datasets (Figure 5A and Figure 6A), the recorded ΔOD kinetics at the selected probe wavelengths provide a reasonable estimate of the S_1_ formation rates.

While the time-resolved ΔOD spectra for Chl *a* (Figure 5B) are rather diffuse and structureless, consisting of a single ∆OD band maximum located at ~465 nm, the ∆OD spectra shown in Figure 6B for BChl *a* are relatively more structured and composed of multiple distinct absorption maxima at ~525 nm and ~650 nm. The absorption intensity for BChl *a* (Figure 6B) appears to increase in the spectral region below ~440 nm, but this is outside the stability region of the probe supercontinuum. We attribute these broad ∆OD bands, for both photosynthetic pigments, to excited-state absorption (singlet-singlet) transitions from the S_1_ state. The ultrafast formation kinetics shown in Figure 5C,D for Chl *a* demonstrate that its S_1_ state is occupied with a characteristic time constant of ~180 ± 74 fs, and the corresponding spectral changes essentially persist for hundreds of picoseconds following optical excitation, as shown in Figure 5B. We also observed that the equivalent S_1_ formation kinetics for BChl *a* are comparable to those recorded for Chl *a*, as shown in Figure 6C,D. In particular, the ~525 nm and ~650 nm ΔOD band maxima for BChl *a* are formed with time constants of ~215 ± 81 fs and ~351 ± 44 fs, respectively. This slight difference in the respective formation kinetics of the absorption maxima for BChl *a* may be due to the inherent variation of the pump–probe cross-correlation function at different probe wavelengths [60,61]. As shown in Figure 6B, the excited-state absorption bands for BChl *a* similarly persist for hundreds of picoseconds following optical excitation. For both photosynthetic pigments, we interpret these time constants to be largely indicative of the timescale for ultrafast internal conversion from the B (Soret) to Q energy bands following pulsed excitation at ~400 nm. Because the recorded time constants are comparable to the pump pulse width of ~150 fs, we consider these estimated values as simply upper bounds on the timescale of B→Q internal conversion.

### 3.2. Picosecond Transient Absorption Spectra and Kinetics

**Figure 7:**
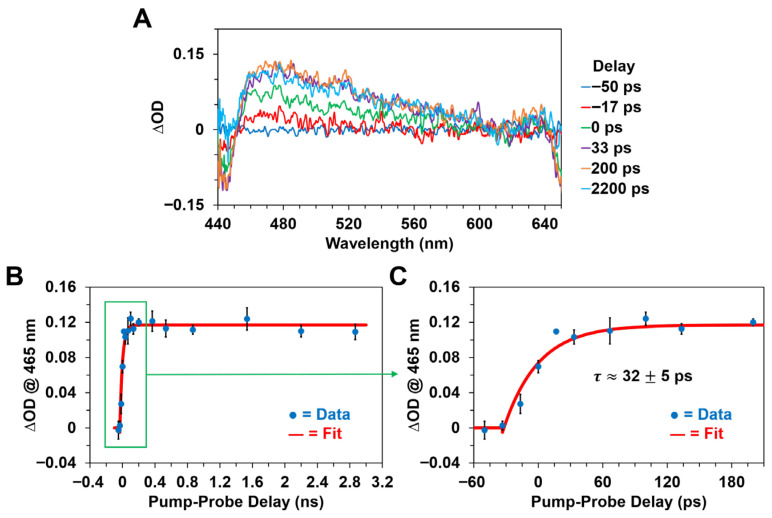
Picosecond transient absorption spectra and kinetics of Chl *a* in methanol following optical excitation with an ~650 nm, ~30 ps pulse with ~1 mJ pulse energy. (**A**) Picosecond transient absorption spectra at various pump–probe delay times. (**B**) Excited-state formation kinetics monitored at the ~465 nm ∆OD band maximum, illustrating the (**C**) ultrafast formation of the first excited singlet state within the picosecond pump pulse duration. Note that the error bars at each data point correspond to one standard deviation from the mean.

**Figure 8:**
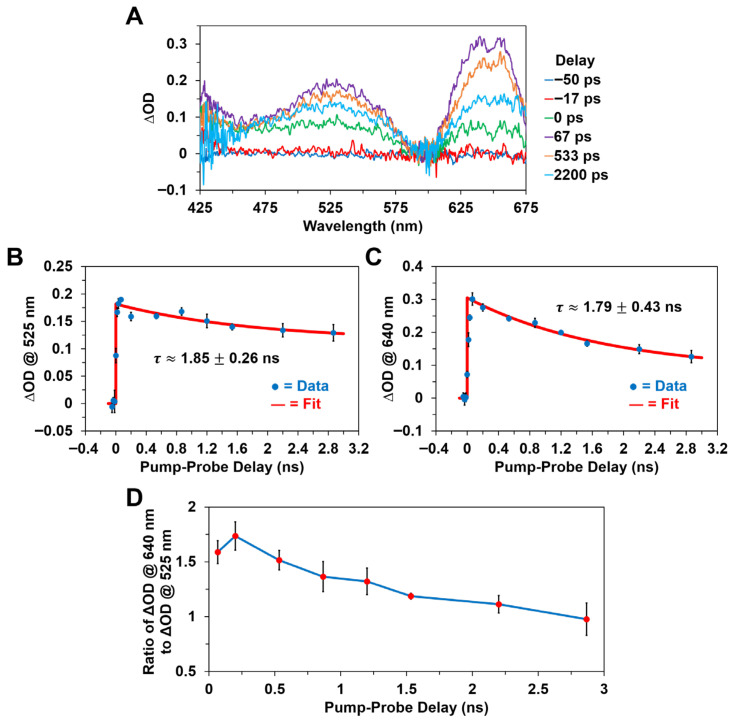
Picosecond transient absorption spectra and kinetics of BChl *a* in methanol following optical excitation with an ~650 nm, ~30 ps pulse with ~1 mJ pulse energy. (**A**) Picosecond transient absorption spectra at various pump–probe delay times. Excited-state decay kinetics monitored at the (**B**) ~525 nm and (**C**) ~640 nm ∆OD band maxima, which demonstrate the ultrafast formation of the first excited singlet state, within the picosecond pump pulse duration, followed by excited singlet-state relaxation on the nanosecond timescale. (**D**) Ratio of the ~640 nm to ~525 nm ΔOD band maxima as a function of pump–probe delay time. For plots (**B**–**D**), note that the error bars at each data point correspond to one standard deviation from the mean.

Figure 7 and Figure 8 depict the picosecond transient absorption spectra and kinetics of Chl *a* and BChl *a* in methanol, respectively, following optical excitation with an ~650 nm, ~30 ps pulse. As expected, the ultrafast transient absorption bands observed following femtosecond pulsed excitation (Figure 5 and Figure 6) are formed practically within the picosecond pump pulse duration for both photosynthetic pigments, as shown in Figure 7 and Figure 8. The recorded picosecond transient absorption spectra are essentially identical, with respect to both the ∆OD band maxima and overall band shapes, to the previously discussed femtosecond time-resolved ΔOD spectra and are thus similarly assigned to the first excited singlet states of the pigments.

Interestingly, we observed that while the transient absorption spectra and ~465 nm ΔOD band maximum for Chl *a* remain practically constant (Figure 7A,B) over the ~3 ns duration of the picosecond experiments, the BChl *a* excited-state absorption band maxima at ~525 nm and ~640 nm gradually decay on the nanosecond timescale, as evidenced in Figure 8B,C, with a characteristic time constant of ~1.8 ns. We assign this observed nanosecond decay to primarily intersystem crossing between the BChl *a* first excited electronic singlet and triplet energy states, S_1_→T_1_, which occurs due to the absence of a suitable acceptor or quencher for the excited singlet state energy of the BChl *a* pigment. While relaxation of the S_1_ state can also occur through non-radiative internal conversion or fluorescence emission to the ground electronic singlet state, S_0_, previous studies [20,69] have demonstrated that intersystem crossing is the dominant mechanism of depopulation for the S_1_ state in photosynthetic pigments like BChl *a*. This is supported by the fact that the ΔOD decay kinetics shown in Figure 8B,C are well fitted by single-exponential models. In addition, the characteristic ΔOD spectral changes accompanying the intersystem crossing transition for BChl *a* are displayed in both Figure 8A,D, which clearly show that the ~640 nm to ~525 nm absorption intensity ratio progressively decreases in time. As noted previously, we did not observe an equivalent decay in the ~465 nm ∆OD band maximum of the Chl *a* pigment over the same experimental timescale. Hence, the picosecond transient absorption data essentially suggests a faster S_1_ relaxation rate for the BChl *a* pigment, likely due in part to more efficient intersystem crossing, while both pigments display ∆OD bands that persist for several nanoseconds following optical excitation with a picosecond pulse. This proposition appears to be consistent with more recent measurements [45] that have demonstrated a lower triplet excited-state (T_1_) yield for Chl *a* than previously reported.

### 3.3. Nanosecond Transient Absorption Spectra and Kinetics

**Figure 9:**
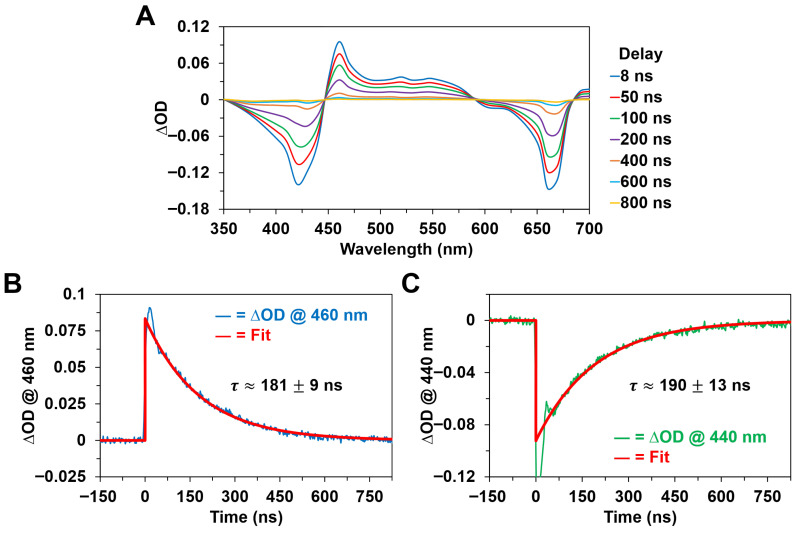
Nanosecond transient absorption spectra and kinetics of Chl *a* in methanol following optical excitation with an ~425 nm, ~8 ns pulse with ~2 mJ pulse energy. (**A**) Nanosecond transient absorption spectra at various pump–probe delay times. These spectra are obtained from the exponential fittings of the ΔOD kinetic traces at each of the detected probe wavelengths. (**B**) Nanosecond ΔOD kinetic trace recorded at a probe wavelength of 460 nm, corresponding to the decay of the first excited triplet state. (**C**) Nanosecond ΔOD kinetic trace recorded at a probe wavelength of 440 nm, corresponding to the repopulation of the ground singlet state. The large-amplitude, rapid decay component in the first ~50 ns after excitation is attributed to scattering of the ~425 nm pump beam.

**Figure 10:**
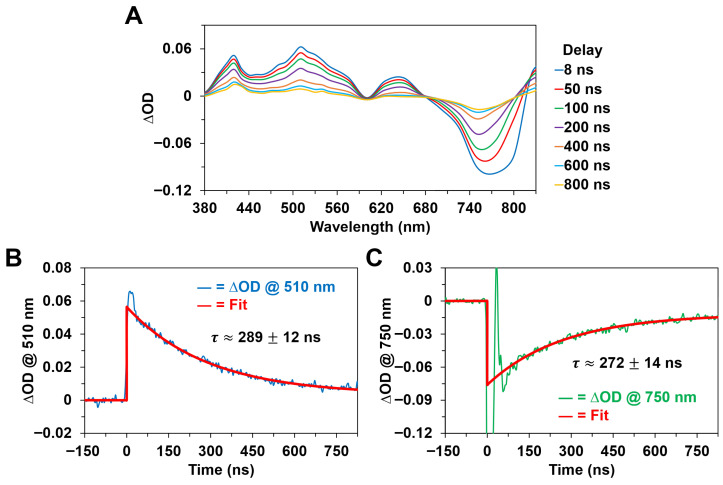
Nanosecond transient absorption spectra and kinetics of BChl *a* in methanol following optical excitation with an ~355 nm, ~8 ns pulse with ~8 mJ pulse energy. (**A**) Nanosecond transient absorption spectra at various pump–probe delay times. These spectra are obtained from the exponential fittings of the ΔOD kinetic traces at each of the detected probe wavelengths. (**B**) Nanosecond ΔOD kinetic trace recorded at a probe wavelength of 510 nm, corresponding to the decay of the first excited triplet state. (**C**) Nanosecond ΔOD kinetic trace recorded at a probe wavelength of 750 nm, corresponding to the repopulation of the ground singlet state. The large-amplitude, rapid decay component in the first ~50 ns after excitation is attributed to fluorescence emission from the first excited singlet state.

Figure 9 and Figure 10 show the nanosecond transient absorption spectra and kinetics of Chl *a* and BChl *a* in methanol, respectively, following optical excitation with ~8 ns pump pulses in their respective Soret absorption bands. In agreement with past studies [13,14,22,23,42,45] on the triplet states of photosynthetic pigments, both Chl *a* and BChl *a* exhibited broad, diffuse ∆OD bands formed within the nanosecond pump pulse duration that are assigned to excited-state absorption (triplet-triplet) transitions from the first excited electronic triplet energy state, T_1_, which is populated by means of intersystem crossing from the first excited singlet state. This is supported by the nanosecond time-resolved spectral data shown in Figure 9A and Figure 10A, which clearly demonstrate that the nanosecond ΔOD spectra for each pigment differ considerably in their respective band shapes relative to the corresponding ultrafast, femtosecond to picosecond, ΔOD spectra previously discussed. Specifically, we note the significant reduction in bandwidth for the ~460–465 nm ∆OD band maximum of the Chl *a* pigment (Figure 5B, Figure 7A and Figure 9A), where the nanosecond transient absorption spectra are noticeably more structured compared to the ultrafast spectral data. Furthermore, we observed changes in the ratio of ∆OD band maxima for the BChl *a* pigment (Figure 6B, Figure 8A and Figure 10A), where the absorption intensity ratio of the ~640–650 nm ∆OD band magnitude to the ~525 nm ∆OD band magnitude has significantly decreased over the picosecond to nanosecond timescales, which is consistent with the intersystem crossing transition assigned for BChl *a* in the corresponding picosecond time-resolved data (Figure 8D). While we were unable to directly observe intersystem crossing for the Chl *a* pigment with either the picosecond or nanosecond time-resolved systems, owing to experimental limitations, consideration of the previously discussed picosecond data and nanosecond pump pulse duration suggests that intersystem crossing occurs on the ~3–8 ns timescale for Chl *a* following pulsed excitation.

In addition to the distinct ΔOD spectra and band maxima recorded for each photosynthetic pigment, we observed that the excited triplet-state decay kinetics of Chl *a* and BChl *a*, as shown in Figure 9B and Figure 10B, are different. While both pigments display a single-exponential decay of the excited triplet state that is kinetically consistent with the concomitant repopulation of the ground singlet state (Figure 9C and Figure 10C), we found that the Chl *a* triplet-state decay occurs with a characteristic time constant of ~181 ± 9 ns, whereas the BChl *a* triplet-state decay is described by a characteristic time constant of ~289 ± 12 ns. Thus, the triplet-state decay kinetics of Chl *a* were faster under our experimental conditions.

We also note that relatively small, nonzero ΔOD constant offsets were observed at long delay times in the kinetic traces for BChl *a* (Figure 10), implying increased absorption in the ~400–600 nm spectral region and decreased absorption in the ~700–800 nm spectral region following photoexcitation, whereas no such offsets were recorded for Chl *a* (Figure 9). These long-lived ΔOD offsets may indicate partial BChl *a* to BChl *b* conversion under our experimental conditions, in contrast to practically complete ground-state repopulation for Chl *a*, but the recorded triplet-state decay kinetics for Chl *a* and BChl *a* are nonetheless both consistent with singlet oxygen generation being the dominant quenching mechanism [23]. Hence, the difference in the excited triplet-state decay kinetics of Chl *a* and BChl *a* in our study possibly reflects variation in the energy transfer or quenching process with molecular oxygen.

### 3.4. Comparison of Chl a and BChl a Singlet-State and Triplet-State Dynamics

**Figure 11:**
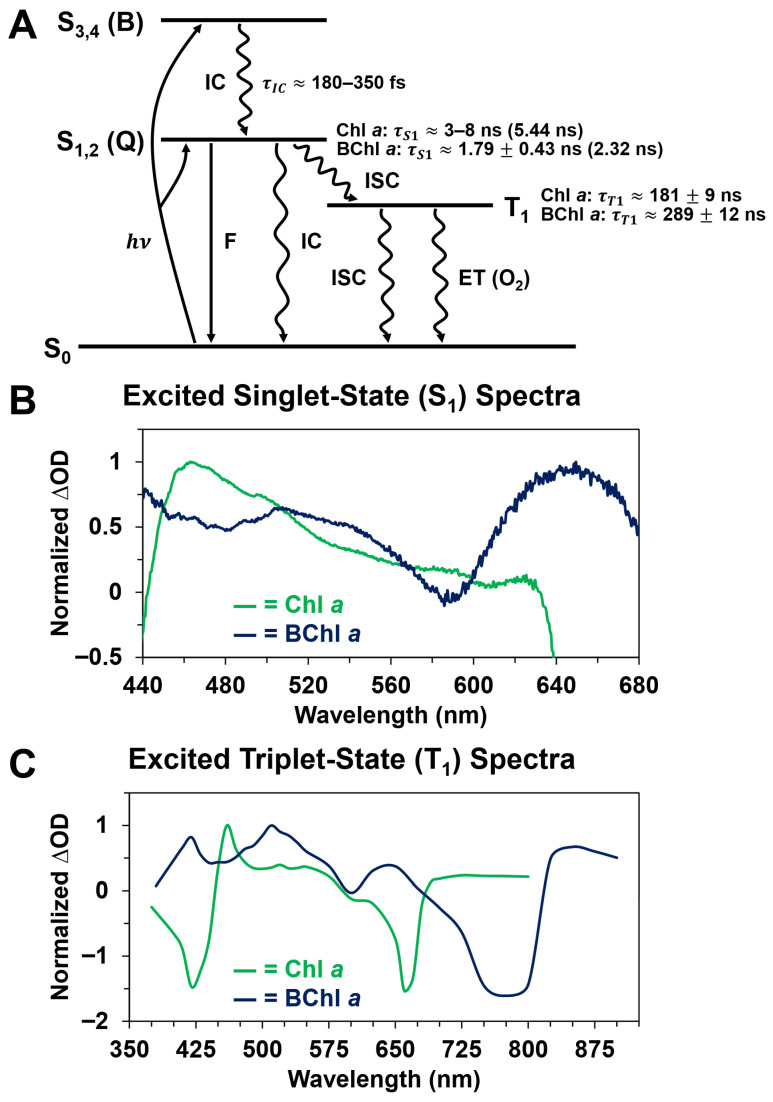
Summary of major spectral and kinetic differences in the ultrafast optical responses of Chl *a* and BChl *a* in methanol. (**A**) Energy level diagram with primary electronic transitions—internal conversion (IC), fluorescence emission (F), intersystem crossing (ISC), and energy transfer (ET) or triplet-state quenching due to interaction with molecular oxygen (O_2_)—between electronic singlet (S) and triplet (T) states, in addition to the corresponding recorded kinetics, noted. The numbers in parentheses for the first excited singlet-state (S_1_) lifetimes are fluorescence lifetime measurements from other studies [19,20] that are provided for comparison with the lifetimes estimated from our transient absorption data. Relaxation from the first excited triplet state (T_1_) to the ground singlet state (S_0_) can occur by both IC and phosphorescence emission [43], but ET to O_2_ is the primary mechanism of depopulation under our experimental conditions. Note that relaxation processes within the B (S_4_→S_3_) and Q (S_2_→S_1_) bands, which are each represented by individual electronic states, are omitted for simplicity. (**B**) Comparison of excited singlet-state (S_1_) normalized ΔOD spectra for both pigments following B (Soret) band excitation. (**C**) Comparison of excited triplet-state (T_1_) normalized ΔOD spectra for both pigments following B (Soret) band excitation.

The ultrafast time-resolved absorption data for Chl *a* and BChl *a*, as summarized in Figure 11, show several interesting similarities and differences in the respective optical responses of these two distinct types of photosynthetic pigments. Similarities include the ultrafast formation of excited-state absorption bands in the visible spectral region on the femtosecond timescale (Figure 5 and Figure 6), corresponding to the occupation of the first excited electronic singlet energy state for each pigment. These excited singlet-state ΔOD band maxima persisted for several hundreds of picoseconds, which eventually resulted in subsequent intersystem crossing to the first excited electronic triplet energy state for each pigment, within a few nanoseconds, as shown in Figure 7 and Figure 8. Relaxation back to the ground singlet state was then observed within hundreds of nanoseconds after optical excitation (Figure 9 and Figure 10).

In addition to these similarities, various spectral and kinetic differences are also observed between the photosynthetic pigments studied. Notably, the transient absorption spectra for BChl *a* appear to be more complex in their overall shapes, consisting of multiple, distinct ΔOD band maxima with varying bandwidths, whereas the corresponding ∆OD spectra for Chl *a* essentially consist of a single broad and structureless band in the visible spectral region. We also determined that the corresponding formation and decay kinetics of various electronic transitions were different between the pigments. Specifically, while the formation kinetics of the first excited singlet state are comparable between Chl *a* and BChl *a*, as demonstrated in Figure 5 and Figure 6, the subsequent intersystem crossing transition process occurs faster for BChl *a* by a factor of at least ~1.7. Following intersystem crossing, the triplet-state decay kinetics of Chl *a* are faster by a factor of ~1.6, as shown in Figure 9B and Figure 10B. Hence, there are significant spectral and kinetic differences between these photosynthetic pigments on the femtosecond to sub-microsecond timescales.

Our ultrafast time-resolved absorption data essentially demonstrates an interplay between the photosynthetic pigment type and corresponding optical response, which results in interesting spectral and kinetic differences on the femtosecond to sub-microsecond timescales. We attribute these differences to the inherent structural modifications (Figure 1A,B) that distinguish the chlorin-type (Chl *a*) and bacteriochlorin-type (BChl *a*) photosynthetic pigments, namely with respect to the differing terminal substituents and degrees of conjugation in the pigment macrocycle [2]. While previous comparative studies [23,42,43,47,49,51,71] have primarily addressed steady-state and excited singlet-state or triplet-state properties on the nanosecond and longer timescales, the time-resolved experimental results in our study provide additional perspective on how ultrafast processes, such as the B→Q internal conversion (Figure 5 and Figure 6) and S_1_→T_1_ intersystem crossing (Figure 7 and Figure 8) transitions, in photosynthesis are affected by alterations in the photosynthetic pigment structure. This finding is consistent with a recent study that examined the effects of varying terminal substituents on the excited singlet-state lifetimes of bacteriochlorin-type pigments [72]. In addition to these ultrafast properties, the observed differences in the excited triplet-state decay kinetics (Figure 9 and Figure 10), which are largely influenced by variation in the degree of quenching due to singlet oxygen production [23,43], further characterize the photosynthetic pigment relaxation dynamics following photon absorption. The transient absorption measurements presented in this study help characterize spectral changes and properties associated with excited singlet-state and triplet-state relaxation that are inaccessible through fluorescence or phosphorescence lifetime measurements alone. Such information is important for understanding the mechanisms of both natural and artificial photosynthetic systems that employ similar photosynthetic pigments for light absorption and energy harvesting.

A major limitation of our present study is that we have compared the spectral and kinetic properties of Chl *a* and BChl *a* for only a single solvent. While methanol has been readily used in previous experimental [19,20,21,23,73,74,75,76,77,78,79,80] and theoretical [81,82,83] spectroscopic studies of Chl *a* and BChl *a* pigments, the specific pigment–solvent interactions involved are highly complex. These can result in partial degradation and generate multiple derivative products [84,85,86,87,88,89,90,91,92] with possibly distinct spectroscopic properties, thus complicating spectral analysis. The coordination form of the pigment can also be varied in the solvent [93,94]. We find that our recorded steady-state (Figure 1C,D) and time-resolved (Figure 11B,C) spectra are consistent, with respect to the characteristic band shapes and intensity maxima, with previous studies conducted on Chl *a* [23,42,51,68] and BChl *a* [42,47,69,95] with methanol and other organic solvents. This suggests that we are still capturing the major spectral and kinetic properties associated with the primary chlorin-type (Chl *a*) and bacteriochlorin-type (BChl *a*) structures, even if partial degradation is occurring. To more conclusively identify the structural basis for our spectroscopic and kinetic data, the effect of solvent must be more thoroughly characterized in future studies. Our experimental results thus motivate additional ultrafast studies of chlorin-type and bacteriochlorin-type photosynthetic pigments in various solvents to investigate whether similar spectral and kinetic trends occur.

In future work, it will be of particular interest to conclusively determine how the B→Q energy transfer rates quantitatively compare on the femtosecond timescale among different photosynthetic pigments. A previous computational and experimental study [50] has shown that the B→Q internal conversion rate is slightly faster for Chl *a* compared to Chl *b*, with the two chlorin-type pigments differing by only a single substituent attached to the pigment macrocycle [2]. Additionally, polarized 2D electronic spectroscopy has been applied to study the kinetics of S_2_→S_1_ internal conversion within the Q band for Chl *a* and BChl *a*, and it was determined that Chl *a* relaxes faster [52]. Various other theoretical and experimental studies have also investigated the mechanism and kinetics of ultrafast non-radiative relaxation within the Q band for photosynthetic pigments [47,51,67,80,96,97]. With our current pump–probe temporal resolution and experimental error, we cannot definitively conclude at this time whether the B→Q internal conversion rate is faster for Chl *a* or BChl *a*. The recorded relaxation lifetimes of ~180–350 fs (Figure 11), which are likely pulse-limited for both pigments, agree well with the general timescale for internal conversion observed in several related computational and experimental studies [50,70,80,96,98]. It is worth noting, though, that relatively few experimental ultrafast studies [70,98,99] on photosynthetic pigments have specifically addressed the excited-state dynamics of B (Soret) band excitation, particularly in protic solvents like methanol. Thus, our study provides a preliminary upper bound and comparison of the B→Q internal conversion rates for Chl *a* and BChl *a* in identical solvents, which advances knowledge about their electronic structures.

While we have compared the transient absorption spectra and kinetics of only electronic transitions between Chl *a* and BChl *a* in this study, it will also be important in future studies to characterize the respective vibrational structures of these and related photosynthetic pigments. Advanced data processing techniques, such as global and target analysis [66,100], have been utilized to reveal the ultrafast dynamics of molecular relaxation processes such as intramolecular vibrational redistribution (IVR), intermolecular vibrational cooling, and solvent dielectric relaxation, all of which mediate the dissipation of excited-state energy following photon absorption [21,47,51,70,101]. We intend to employ such analytical techniques in our forthcoming pump–probe studies to compare the vibrational structures, in addition to electronic structures, of different photosynthetic pigments like Chl *a* and BChl *a*. This spectroscopic information will fundamentally advance our understanding of the mechanism of photosynthesis, namely how structural modifications among photosynthetic pigments tune the corresponding spectral and kinetic properties of their excited states that participate in both energy and charge transfer.

## 4. Conclusions

Ultrafast, femtosecond to sub-microsecond, time-resolved absorption spectroscopic measurements were performed on chlorin-type (Chl *a*) and bacteriochlorin-type (BChl *a*) photosynthetic pigments at room temperature to characterize and compare the transient spectra and kinetics of their excited electronic states following photon absorption. Both similarities and differences were observed in the time-resolved spectroscopic and kinetic data, which suggest an interplay between the photosynthetic pigment type, structure, and corresponding excited singlet-state and triplet-state dynamics. Additional ultrafast studies on the distinct vibronic structures of these and other related photosynthetic pigments will further resolve the effects of structural modifications on ultrafast processes in photosynthesis.

## Data Availability

All data are either cited and/or included in the main text.
